# Electrocardiographic and echocardiographic profiles of white-lipped peccaries (*Tayassu pecari*) under ketamine–dexmedetomidine or ketamine–midazolam anesthesia

**DOI:** 10.3389/fvets.2026.1803243

**Published:** 2026-05-29

**Authors:** Rochelle Gorczak, Marilia Avila Valandro, Helena Bulhões Barbosa, Raquel Vieira Niella, Mário Sérgio Lima de Lavor

**Affiliations:** 1 Department of Veterinary Medicine, Universidade Estadual de Santa Cruz, Ilhéus, Bahia, Brazil; 2 Independent Researcher, Porto Alegre, Brazil; 3 Department of Veterinary Medicine, Centro Universitário Ritter dos Reis, Porto Alegre, Rio Grande do Sul, Brazil

**Keywords:** cardiology, dissociative anesthesia, heart, hemodynamics, Tayassuidae, wild mammal

## Abstract

White-lipped peccaries (*Tayassu pecari*) are key frugivores in Neotropical ecosystems and are classified as a vulnerable species, highlighting the need for safe, evidence-based anesthetic protocols for clinical management and conservation actions. To the best of our knowledge, this is the first study to investigate the effects of different dissociative anesthetic protocols on electrocardiographic and echocardiographic parameters in white-lipped peccaries, as well as to establish preliminary cardiovascular reference values for the species. Sixteen clinically healthy adult males, with a mean body weight of 30.7 kg, were evaluated and subjected to anesthesia with ketamine combined with midazolam administered intramuscularly (IM) or ketamine combined with dexmedetomidine administered either IM or intravenously (IV). Electrocardiographic (ECG) monitoring was performed for 30 min to assess mean heart rate (HR), waveform morphology, and the duration of waves and intervals. Echocardiography (ECHO) was used to measure ventricular dimensions, interventricular septal and myocardial wall thicknesses, as well as left atrial and aortic dimensions. The IM dexmedetomidine protocol resulted in a significant reduction in P wave amplitude compared to the midazolam protocol (*p* = 0.016), whereas IV administration was associated with a lower T wave amplitude compared to IM dexmedetomidine (*p* = 0.01) and a lower mean HR when compared to the midazolam group (*p* = 0.03), in addition to the occurrence of supraventricular and ventricular arrhythmias and second-degree atrioventricular block. No morphological cardiac abnormalities were detected on ECHO; however, a significant reduction in interventricular septal thickness during diastole was observed in the IM dexmedetomidine group (*p* = 0.01), without statistically significant changes in quantitative parameters. These findings provide the first integrated ECG and ECHO reference values for *Tayassu pecari*, supporting safer anesthetic decision-making, improved clinical management, and evidence-based conservation strategies.

## 1 Introduction

White-lipped peccaries (*Tayassu pecari*) are mammals of the family Tayassuidae widely distributed throughout Neotropical regions, ranging from southeastern Mexico to northern and central South America. The species plays a fundamental ecological role as a frugivore, contributing to seed dispersal, forest regeneration, and the maintenance of ecosystem structure and functionality (1–4). Despite this relevance, *T. pecari* is currently classified as a vulnerable species due to population declines associated with habitat loss and fragmentation, hunting, and competition with wild boars, with prediction indicating an even more pronounced reduction in upcoming generations (3, 5). In contrast, captive populations exhibit high reproductive rates, which often require clinical interventions and reproductive management (1, 3).

Reports of deaths due to stress associated with physical restraint, resulting in capture myopathy, have been described (44, 45). In this context, chemical restraint becomes indispensable to ensure the safety of both animals and personnel involved in clinical and management procedures. Several anesthetic protocols have been described for the species, with particular emphasis on combinations of dissociative agents and α2-adrenergic agonists, most notably ketamine combined with xylazine (4, 6–12).

More recently, dexmedetomidine has gained prominence in veterinary medicine due to its greater selectivity, potent sedative effects, and the possibility of pharmacological reversal, characteristics that are particularly relevant for the management of wildlife species (13). However, the cardiovascular effects of these drugs in *T. pecari* remain poorly characterized.

*α*₂-adrenergic agonists, such as dexmedetomidine, significantly influence cardiovascular and autonomic responses during anesthesia, modulating anesthetic depth and electrophysiological parameters (14, 15). These agents are known to affect heart rate regulation and may be associated with arrhythmogenic events due to their effects on the balance between sympathetic and parasympathetic tone. Such perioperative physiological variability is closely related to pharmacological interactions, reflecting the complexity of anesthetic management, particularly in wildlife species. However, extrapolation of cardiovascular findings from domestic pigs to wild peccaries should be approached with caution due to important differences in genetics, stress response, and environmental adaptation.

Cardiovascular assessment is a fundamental component of anesthetic monitoring. Electrocardiography (ECG) is a widely used non-invasive method for evaluating cardiac electrical activity, allowing the identification of rhythm disturbances, conduction abnormalities, and alterations in myocardial repolarization (16–18). Given that *α*₂-adrenergic agonists are associated with clinically relevant cardiovascular effects, ECG monitoring during their administration is recommended (13, 19), with these references based on literature reviews involving canines, felines, equines, ruminants, domestic swine, and humans. Despite this, there are no systematic descriptions of ECG parameters for white-lipped peccaries, either under basal conditions or during anesthesia.

Complementarily, echocardiography (ECHO) is the primary non-invasive method for assessing cardiac morphology and function, enabling the evaluation of chamber dimensions, wall thickness, and valvular dynamics (20, 21). Although its applicability has been demonstrated in collared peccaries, a species within the same family (7), to date there are no echocardiographic studies described for *T. pecari*, representing a relevant gap in clinical management and conservation strategies.

While ECG and ECHO reference values are well established for domestic species (22), there is a marked scarcity of cardiovascular data for wildlife species, particularly tayassuids (6, 8, 12), and it is important to emphasize that establishing true reference values in free-ranging wildlife is inherently unfeasible due to biological and environmental variability. In this context, the present study aimed to establish, for the first time, preliminary ECG and ECHO reference values in adult, clinically healthy male white-lipped peccaries (*Tayassu pecari*) under dissociative anesthesia with ketamine combined with dexmedetomidine or midazolam, as well as to characterize the effects of these protocols on the evaluated cardiovascular variables.

## 2 Methods

### 2.1 Animals and ethics

Sixteen white-lipped peccaries (*Tayassu pecari*), adult males, clinically healthy, with a mean body weight of 30.7 kg, were included in the study. The animals were subjected to orchiectomy as a population control measure. The study was conducted at the Fundação Zoobotânica do Rio Grande do Sul, at the Sapucaia do Sul Zoological Park, and was approved by Animal Ethics Committee (CEUA/UESC no. 010/23). All procedures were performed in accordance with national ethical guidelines for the use of wildlife in research.

### 2.2 Study design

On the day prior to the anesthetic–surgical procedure, the animals were separated from their social groups and housed in holding pens within the transition sector, with up to three individuals per pen. Transfer to the enclosures was performed using food-based stimuli, minimizing handling-related stress. After allocation, the animals were fasted for approximately 12 h. Specimen selection was performed randomly by draw, and the exclusion of females was confirmed by visual inspection of the reproductive organs.

Handling was initiated during the early morning hours in order to reduce thermal and physical stress associated with increasing ambient temperatures. Initially, body weight was visually estimated, and this estimation was used as the basis for calculating the doses to be administered intramuscularly (IM): ketamine at 8 mg/kg, dexmedetomidine at 25 μg/kg, and midazolam at 0.3 mg/kg. Given the aggressive nature of the species, which precludes safe physical restraint, drugs were administered via projected darts using a remote drug delivery system (Deskinjet®, Brazil).

The animals were assigned to three experimental groups and submitted to dissociative anesthesia with ketamine (8.68 ± 1.4 mg/kg, IM). Group 1 (Q1; *n* = 5) received ketamine combined with midazolam (0.32 ± 0.05 mg/kg, IM). Group 2 (Q2; *n* = 5) received ketamine combined with dexmedetomidine (25.46 ± 1.22 μg/kg, IM). Group 3 (Q3; *n* = 6) received ketamine alone in the IM protocol and, after the animal reached recumbency, dexmedetomidine (25 μg/kg) was administered intravenously (IV), diluted in sterile saline and delivered slowly over two minutes. The administered doses were individually calculated after weighing each animal. Prior to the surgical procedure, local anesthetic blockade was performed by infiltration with 2% lidocaine (4 mg/kg) at the incision line and in the region of the spermatic cord, in order to provide adequate analgesia and minimize nociceptive stimuli, thereby preventing possible hemodynamic changes associated with pain.

### 2.3 Electrocardiographic and anesthetic monitoring study

After sedation, with a mean time of six minutes, and confirmation of safe conditions for physical handling, the animals were positioned on the surgical table. ECG monitoring was performed for a period of 30 min, during which the animals underwent the surgical procedure. Recordings were obtained at a paper speed of 50 mm/s and an amplitude of 20 mm/mV, using equipment specifically designed for veterinary use (InCardioX^®^, InPulse Animal Health, Brazil).

Quantitative measurements were obtained from lead II, whereas cardiac rhythm analysis included leads I, II, III, aVF, aVR, and aVL. Electrodes were placed on the forelimbs, proximal to the olecranon on the caudal aspect, and on the pelvic limbs, proximal to the patella on the medial aspect, as described by Filippi (23). The mean heart rate (HR) was recorded, along with the duration and amplitude of the P wave, QRS complex, and T wave. In addition, the durations of the P–R and QT intervals and the S–T segment were evaluated.

The device software was used to identify arrhythmias and conduction disturbances, as well as to analyze heart rate variability in the time domain using the following variables: the standard deviation of all normal NN intervals (SDNN, ms), the root mean square of successive differences between NN intervals (rMSSD, ms), and the mean NN interval (mean NN, ms).

A parallel study was conducted with comprehensive anesthetic monitoring. The variables collected included systolic arterial pressure, measured using the oscillometric method with the cuff positioned on the pelvic limb. Respiratory rate was determined by observing thoracic movements, while peripheral oxygen saturation was monitored using a digital oximeter attached to the animals’ snout. Body temperature was measured at the beginning and at the end of the procedure using a digital thermometer inserted into the rectal ampulla.

### 2.4 Echocardiographic study

**Figure 1:**
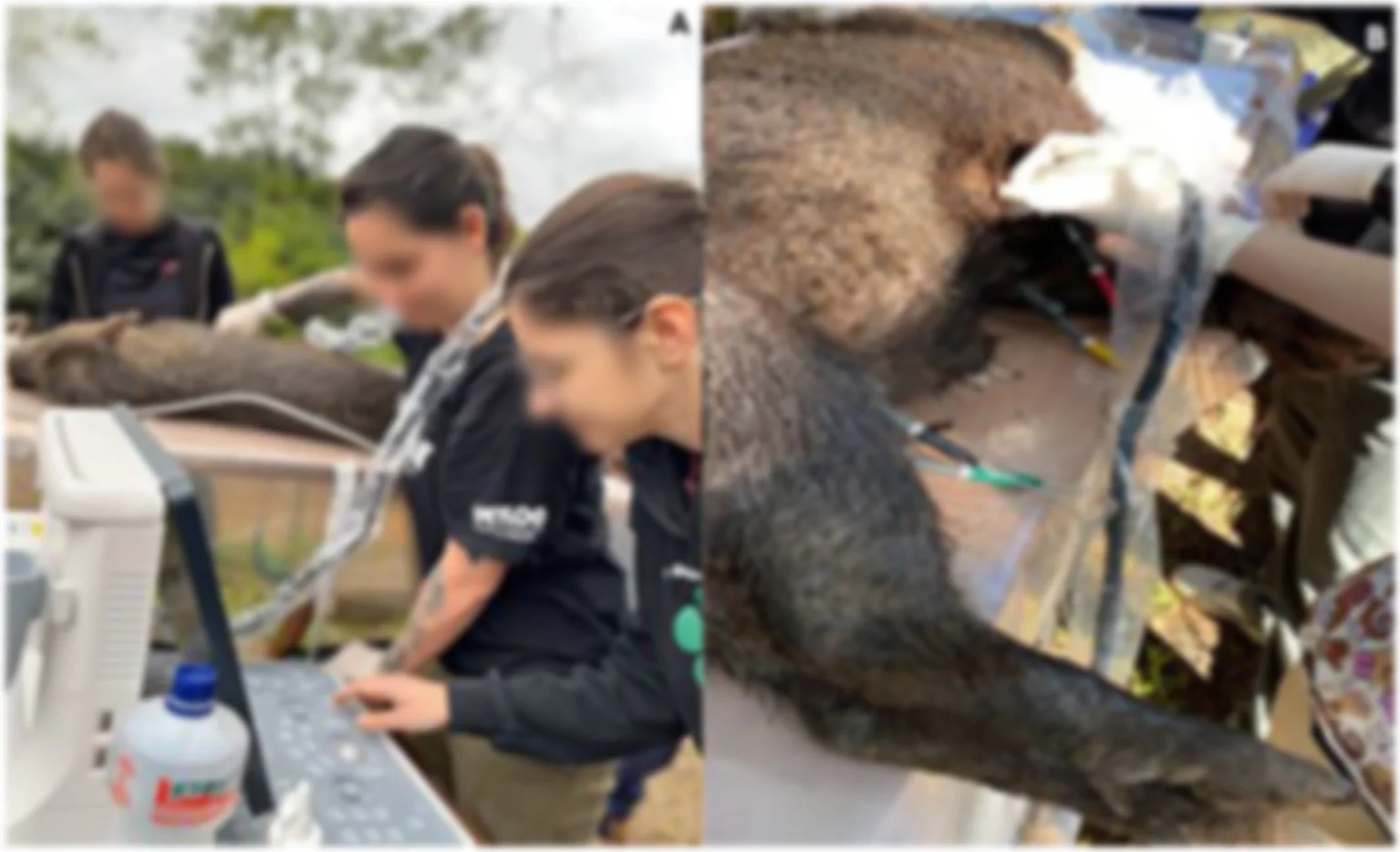
Performance of echocardiography in white-lipped peccaries (*Tayassu pecari*). **(A)** Examiner positioning during the procedure using a Z60 Vet^®^ (Mindray). **(B)** Animal positioned in lateral recumbency with a phased-array probe, using the parasternal window for image acquisition. Source: Personal archive (2023).

ECHO evaluation was performed prior to the surgical procedure, with the animals positioned in left lateral recumbency, using a veterinary ultrasound system (Z60 Vet^®^, Mindray, Brazil). Images were obtained through the right parasternal window, with ultrasound gel applied directly to the thoracic region, without the need for prior hair clipping (Figure 1). A phased-array transducer was used, and images were acquired in M-mode.

The following parameters were measured: left ventricular internal diameter in diastole (LVIDd) and systole (LVIDs); right ventricular internal diameter in diastole (RVIDd); interventricular septal thickness in diastole (IVSd) and systole (IVSs); left ventricular posterior wall thickness in diastole (LVPWd) and systole (LVPWs); and right ventricular free wall thickness in diastole (RVFWd). In addition, aortic diameter (Ao), left atrial diameter (LA), and the left atrium-to-aorta ratio (LA/Ao) were evaluated.

All cardiological assessments were performed by the same board-certified veterinary cardiologist to minimize interobserver variability.

### 2.5 Statistical analysis

The experimental design was completely randomized. Data were initially assessed for normality prior to statistical analysis. Parametric data were analyzed using analysis of variance (ANOVA) followed by the Bonferroni test. Additionally, pairwise comparisons between groups (Q1 vs. Q2, Q2 vs. Q3, and Q1 vs. Q3) were performed using the unpaired Student’s t-test. Results were expressed as mean ± standard error of the mean (SEM). A significance level of 5% (*p* < 0.05) was adopted for all analyses.

## 3 Results

### 3.1 Electrocardiographic analysis and anesthetic monitoring

Throughout the ECG monitoring period, most of the evaluated variables did not show statistically significant differences among the anesthetic protocols. However, isolated differences were observed between groups. The P-wave amplitude was significantly lower in group Q2 (ketamine + dexmedetomidine IM) compared with group Q1 (ketamine + midazolam IM) (0.06 ± 0.02 mV vs. 0.13 ± 0.03 mV; *p* = 0.016). In addition, the T-wave amplitude was significantly lower in group Q3 (ketamine IM + dexmedetomidine IV) compared with group Q2 (−0.09 ± 0.14 mV vs. −0.37 ± 0.12 mV; *p* = 0.010).

Heart rate variability analysis demonstrated a significant increase in the mean NN interval (mean NN) in group Q3 compared with group Q1 (751.7 ± 69.0 ms vs. 587.0 ± 97.4 ms; *p* = 0.032). No statistically significant differences were identified among groups for the remaining electrocardiographic variables, including heart rate, wave and interval durations, cardiac axis, SDNN, and rMSSD.

Given the absence of significant differences in most parameters, data were pooled, allowing the establishment of representative preliminary ECG reference values for the species (Table 1).

**Table 1:** Electrocardiographic variables of white-lipped peccaries (*Tayassu pecari*) subjected to dissociative anesthesia (mean ± SEM) (paper speed: 50 mm/s; amplitude: 20 mm/mV).

Variable	Q1	*p* value	Q2	*p* value	Q3	*p* value	Confidence interval	Overall mean ± SEM
HR (bpm)	103.6 ± 16.28	0.439	91 ± 16.62	0.749	84 ± 14.60	0.139	69–120	92.31 ± 4.22
P wave (mV)	0.13 ± 0.03*	0.090	0.06 ± 0.02*	0.016	0.10 ± 0.05	0.340	0.05–0.19	0.1 ± 0.01
P wave (ms)	44.8 ± 5.93	0.934	47.6 ± 15.83	0.981	49 ± 13.37	0.846	28–68	47.25 ± 2.94
PR interval (ms)	112 ± 23.70	0.404	127.6 ± 14.24	0.705	136.6 ± 16.81	0.109	88–164	126.12 ± 5.06
QRS complex (ms)	57.2 ± 7.15	0.862	59.6 ± 8.29	0.827	57 ± 6.41	0.999	48–74	57.87 ± 1.71
Q wave (mV)	−0.14 ± 0.06	0.991	−0.15 ± 0.09	0.976	−0.15 ± 0.05	0.997	(−0.31)-(−0.1)	−0.14 ± 0.01
S wave (mV)	−0.01 ± 0.02	0.959	−0.01 ± 0.02	1.000	−0.01 ± 0.02	0.948	(−0.01)-(0)	−0.01 ± 0.005
R wave (mV)	1.34 ± 0.24	>0.99	1.34 ± 0.37	0.195	1.01 ± 0.25	0.198	0.66–1.81	1.22 ± 0.07
QT interval (ms)	266.8 ± 41.43	0.434	298.4 ± 34.21	0.752	315.6 ± 41.15	0.138	230–386	295 ± 10.53
T wave (mV)	−0.22 ± 0.12	0.221	−0.37 ± 0.12*	0.247	−0.09 ± 0.14*	0.010	(−0.48)-(−0.07)	−0.21 ± 0.04
T wave (ms)	81.6 ± 26.01	0.314	105.6 ± 9.31	0.823	90.6 ± 31.74	0.597	54–138	92.5 ± 6.31
Cardiac axis (°)	72.6 ± 5.45	0.982	70.4 ± 5.24	0.940	76.5 ± 10.99	0.860	39–110	73.37 ± 4.50
SDNN (ms)	191.9 ± 75.7	0.462	301.4 ± 237.6	0.913	226.9 ± 49.0	0.669	90.18–719.80	237.35 ± 40.03
rMSSD (ms)	221.9 ± 124.5	0.717	277.4 ± 143.3	0.858	186.2 ± 57.6	0.392	94.12–505.79	226.81 ± 31.74
Mean NN (ms)	587.0 ± 97.4*	0.340	674.3 ± 116.2	0.392	751.7 ± 69.0*	0.032	497.32–863.10	667.56 ± 31.51

Q1 – Ketamine + midazolam IM; Q2 – Ketamine + dexmedetomidine IM; Q3 – Ketamine IM + dexmedetomidine IV. HR – Heart rate; SDNN – Standard deviation of all normal NN intervals over a 24-h recording period; rMSSD – Root mean square of successive differences between NN intervals; mean NN – Mean of all NN intervals in the recording (mean ± SEM; *p < 0.05; unpaired Student’s t-test for comparisons between two groups).

**Figure 2:**
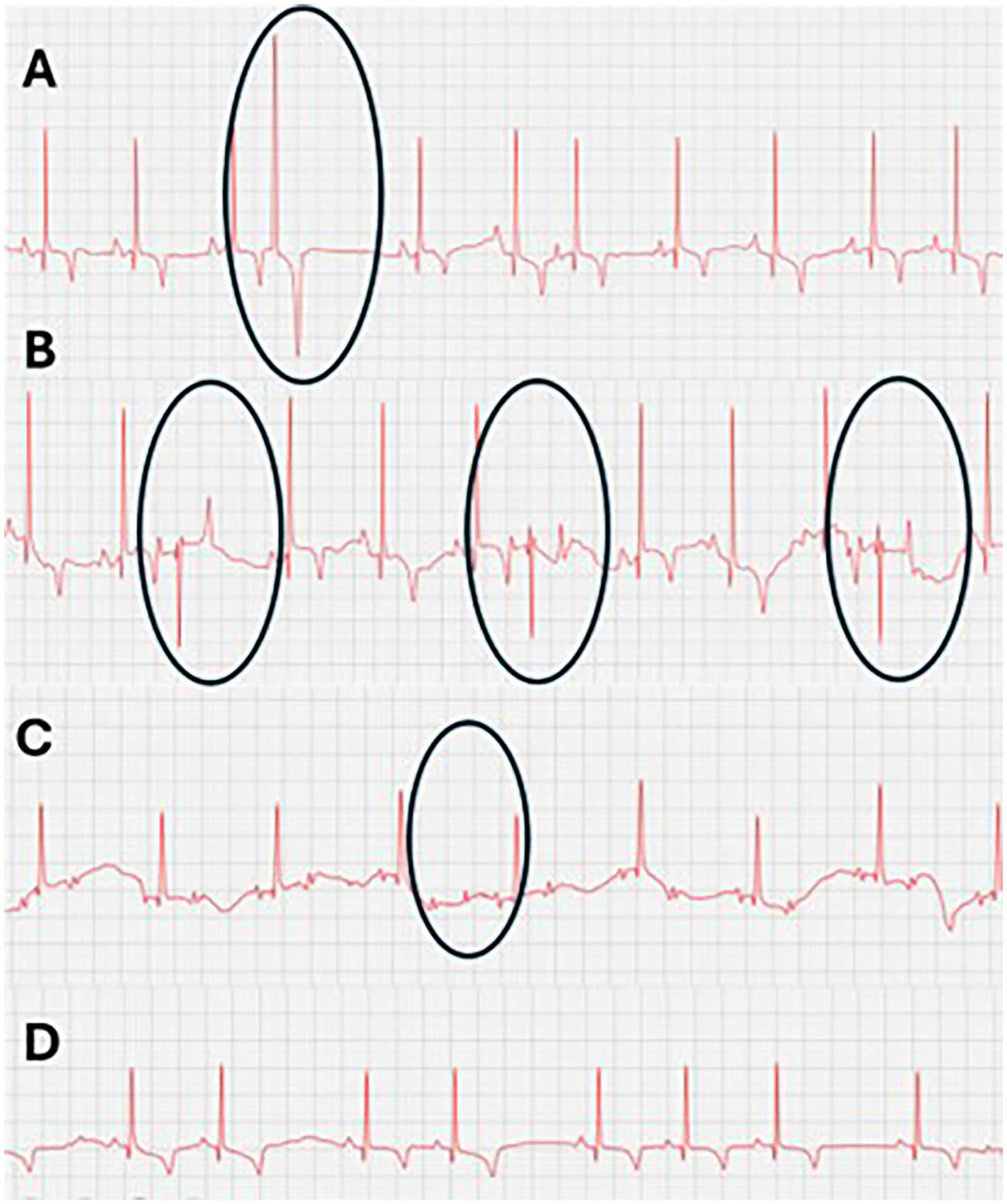
Electrocardiographic tracings (lead II) of white-lipped peccaries (*Tayassu pecari*) under dissociative anesthesia. **(A)** Q3 animal showing isolated premature supraventricular complexes. **(B)** Q3 animal presenting premature ventricular complexes, both isolated and in trigeminy. **(C)** Q3 animal with second-degree atrioventricular block (AV block). **(D)** Q2 animal showing sinus arrhythmia. Alterations are highlighted by a circle. Recording settings: paper speed 50 mm/s and amplitude 20 mm/mV. Q2: ketamine + dexmedetomidine IM; Q3: ketamine IM + dexmedetomidine IV.

Rhythm alterations and conduction disturbances were predominantly observed in group Q3. Two animals in this group presented isolated premature supraventricular complexes (Figure 2A), one of which also exhibited premature ventricular complexes, both isolated and in a trigeminy pattern, unifocal with compensatory pauses (Figure 2B). Additionally, two animals in group Q3 showed second-degree atrioventricular block (AV block), Mobitz type II (Figure 2C). In group Q2, only one animal presented sinus arrhythmia (Figure 2D), whereas no rhythm or conduction abnormalities were observed in group Q1.

At the first measurement, mean systolic arterial pressure (SAP) was 146.6 ± 10.0 mmHg in group Q1, 158.0 ± 19.2 mmHg in group Q2, and 177.1 ± 32.6 mmHg in group Q3. During the evaluated period, Q3 showed significantly higher SAP values compared to groups Q1 and Q2. Regarding body temperature, no significant reductions were observed after the administration of the anesthetic protocols, with mean values of 38.01 ± 0.42 °C in Q1, 38.31 ± 0.62 °C in Q2, and 38.04 ± 0.84 °C in Q3 after 30 min, as recorded.

### 3.2 Echocardiographic analysis

**Figure 3:**
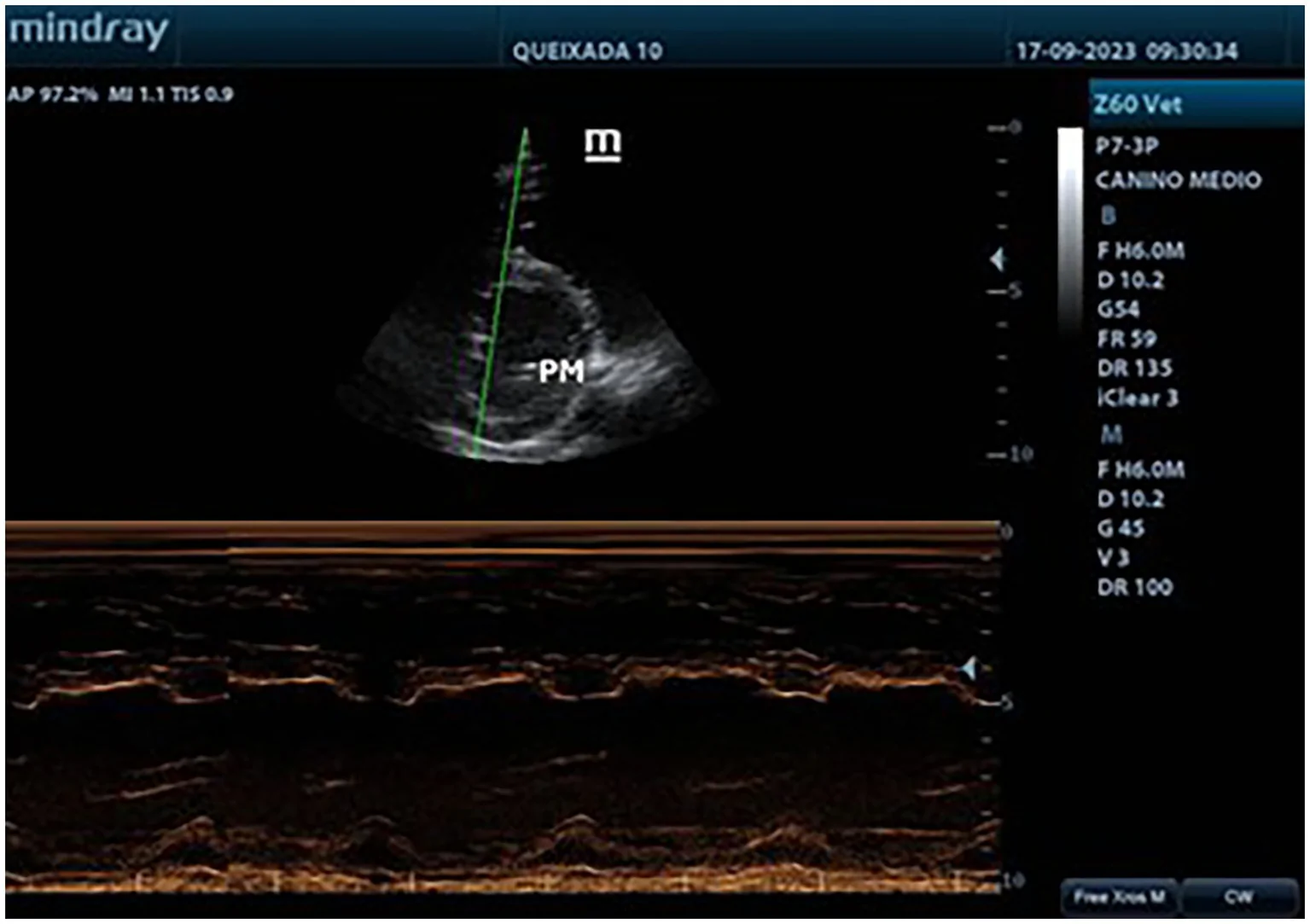
Echocardiographic image of a white-lipped peccary (*Tayassu pecari*) u*n*der dissociative anesthesia obtained from the right parasternal window, short-axis view at the level of the papillary muscle (PM), M-mode.

**Figure 4:**
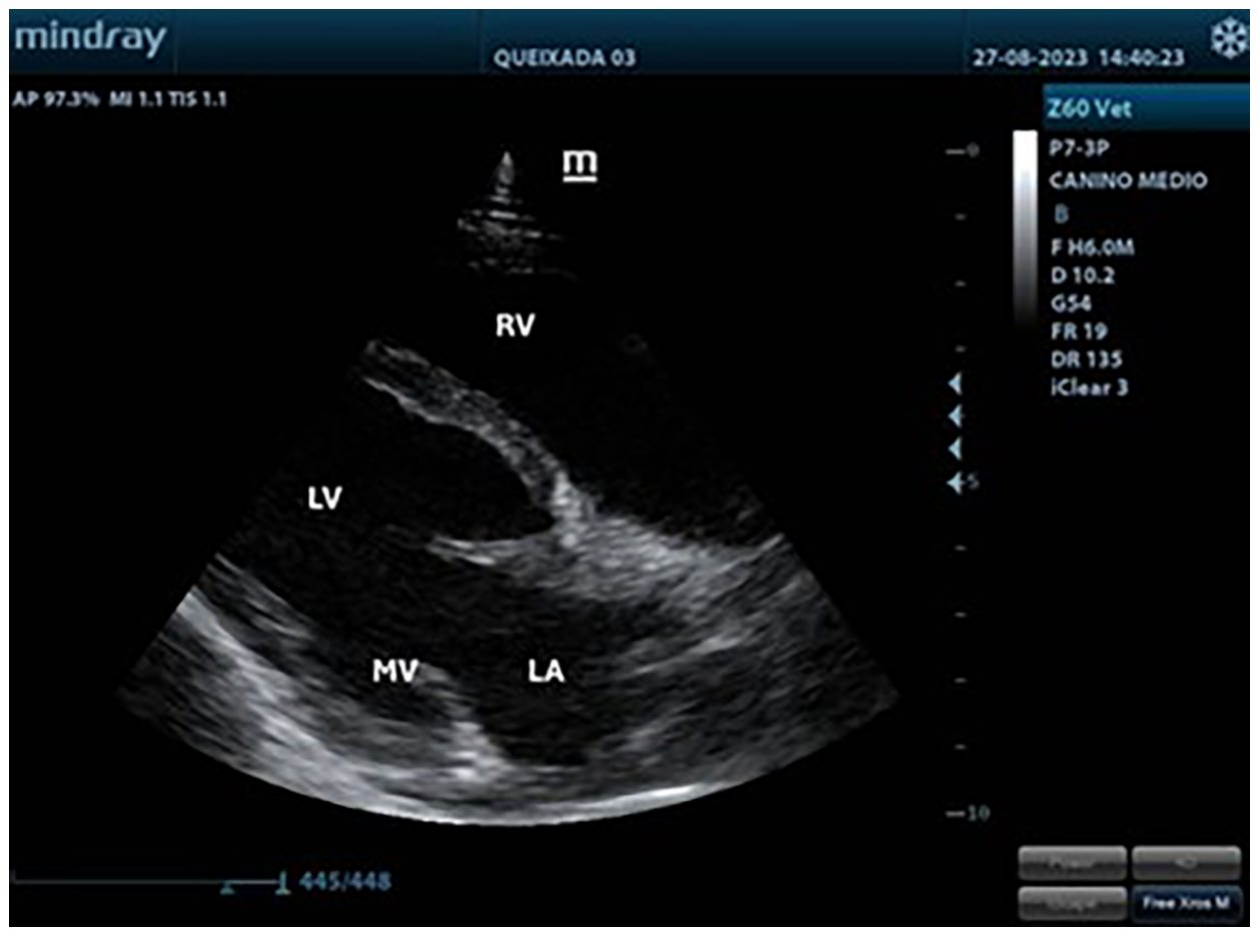
Echocardiographic image of a white-lipped peccary (*Tayassu pecari*) u*n*der dissociative anesthesia obtained from the right parasternal window, long-axis view, showing the morphology of the mitral valve (MV), left atrium (LA), left ventricle (LV), and right ventricle (RV).

Echocardiographic evaluation was performed with the animals positioned in lateral recumbency, allowing ventricular measurements to be obtained in M-mode through the parasternal window. No valvular morphological abnormalities or structural changes in the cardiac chambers were observed in any of the evaluated animals (Figures 3, 4).

Among the measured parameters, a significant reduction in interventricular septal thickness in diastole (IVSd) was observed in group Q2 compared with group Q1 (0.52 ± 0.17 cm vs. 0.85 ± 0.10 cm; *p* = 0.014). The remaining echocardiographic variables did not show statistically significant differences among groups (Table 2). Therefore, the obtained values were pooled to establish preliminary reference intervals for echocardiographic parameters for the species. Fractional shortening (FS) showed values of 54.75, 77.79, and 56.79% in groups Q1, Q2, and Q3, respectively. Similarly, ejection fraction (EF) was 80.6, 47.8, and 78.1% in the respective groups.

**Table 2:** Echocardiographic variables of white-lipped peccaries (*Tayassu pecari*) under dissociative anesthesia (mean ± standard error).

Variable	Q1	*p* value	Q2	*p* value	Q3	*p* value	Confidence interval	Overall mean ± SEM
RVFWd (cm)	0.41 ± 0.08	0.950	0.39 ± 0.05	0.338	0.49 ± 0.17	0.494	0.31–0.76	0.43 ± 0.03
RVIDd (cm)	0.88 ± 0.35	0.992	0.84 ± 0.58	0.266	1.40 ± 0.54	0.223	0.36–1.88	1.04 ± 0.13
IVSd (cm)	0.85 ± 0.10*	0.062	0.52 ± 0.17*	0.014	0.60 ± 0.16	0.677	0.31–0.99	0.66 ± 0.05
LVIDd (cm)	3.16 ± 0.51	0.487	2.67 ± 0.46	0.693	3.50 ± 0.89	0.152	2.01–4.43	3.11 ± 0.18
LVPWd (cm)	1.52 ± 0.61	0.547	1.19 ± 0.52	0.173	0.92 ± 0.26	0.677	0.45–1.93	1.21 ± 0.13
IVSs (cm)	0.79 ± 0.18	0.730	0.70 ± 0.26	0.637	0.91 ± 0.12	0.249	0.45–1.16	0.80 ± 0.05
LVIDs (cm)	1.83 ± 0.02	0.661	2.15 ± 0.60	0.725	2.11 ± 0.81	0.994	1.25–3.13	2.03 ± 0.14
LVPWs (cm)	1.56 ± 0.45	0.294	1.14 ± 0.46	0.784	1.38 ± 0.34	0.649	0.81–1.97	1.36 ± 0.11

Q1 – Ketamine + midazolam IM; Q2 – Ketamine + dexmedetomidine IM; Q3 – Ketamine IM + dexmedetomidine IV. RVFWd—Right ventricular free wall thickness in diastole; RVIDd—Right ventricular internal diameter in diastole; IVSd—Interventricular septal thickness in diastole; LVIDd—Left ventricular internal diameter in diastole; LVPWd—Left ventricular posterior wall thickness in diastole; IVSs—Interventricular septal thickness in systole; LVIDs—Left ventricular internal diameter in systole; LVPWs—Left ventricular posterior wall thickness in systole (mean ± SEM; *p < 0.05; unpaired Student’s t-test for comparisons between two groups).

**Figure 5:**
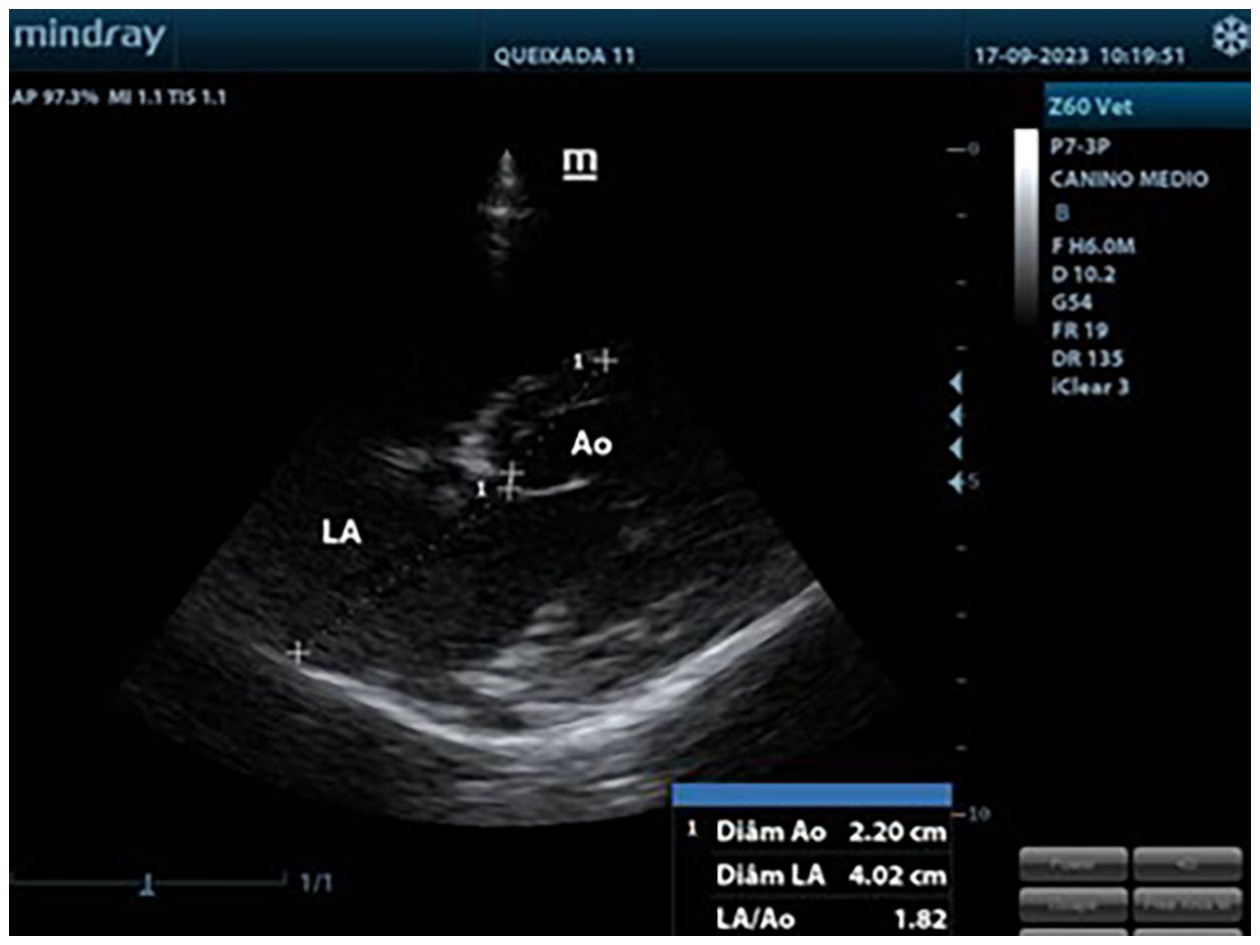
Echocardiographic image of a white-lipped peccary (*Tayassu pecari*) o*b*tained from the right parasternal window, showing the diameter of the left atrium (LA) and the aortic root (Ao).

Additionally, it was possible to measure, in one individual, the left atrial (LA) diameter (4.02 cm), the aortic (Ao) diameter (2.20 cm), and the LA/Ao ratio (1.82), as illustrated in Figure 5.

## 4 Discussion

To our knowledge, this study provides the first preliminary reference ECG e ECHO reference values for clinically healthy, captive adult male white-lipped peccaries (*Tayassu pecari*), addressing a significant gap in the cardiovascular knowledge of this species, which is currently classified as vulnerable. The scarcity of clinical cardiovascular data in white-lipped peccaries is largely attributable to handling challenges and limitations in the application of diagnostic procedures, underscoring the importance of standardizing safe anesthetic protocols and characterizing their cardiovascular effects. Such standardization is essential not only for clinical management but also for the development of evidence-based conservation strategies.

The number of animals included (*n* = 16) is consistent with recommendations for studies aimed at establishing reference values in wildlife species, in which logistical and ethical constraints limit extensive sampling. According to Friedrichs et al. (24), samples composed of at least 10 clinically homogeneous individuals, evaluated using robust analytical methods, allow data to be presented through histograms and described in terms of minimum and maximum values, as well as measures of central tendency such as the mean or median. In such cases, the use of individual-based reference intervals may also be considered. Careful animal selection, standardized procedures, and examinations performed by a single operator contributed to the consistency of the data obtained.

Animals were subjected to anesthetic protocols combining ketamine with either dexmedetomidine or midazolam, and electrocardiographic monitoring was used to detect potential rhythm and conduction abnormalities. ECG is an essential diagnostic tool for identifying arrhythmias and conduction disturbances that may compromise anesthetic safety. It is well recognized that anesthetic agents influence cardiovascular parameters such as heart rate, electrocardiographic wave morphology and amplitude, and conduction interval duration, making ECG monitoring particularly relevant when *α*2-adrenergic agonists such as dexmedetomidine are used (16, 18, 25, 26).

A reduction in heart rate was observed in animals treated with dexmedetomidine (Q2 and Q3) compared with those receiving midazolam (Q1), without characterization of clinically relevant bradycardia, as values remained within physiological limits. Similar findings have been previously reported in white-lipped peccaries subjected to different anesthetic protocols (4, 27), as well as in other species receiving dexmedetomidine–ketamine combinations (28, 29). In collared peccaries, Silva et al. (30) also reported heart rate reduction with dexmedetomidine, highlighting its greater sedative efficacy and lower hypertensive effect compared with detomidine.

The less pronounced decrease in heart rate observed may be attributed to the synergistic interaction between ketamine and dexmedetomidine (13, 29, 31). Although dexmedetomidine exerts central sympatholytic effects mediated by α₂-adrenergic receptors, the initial decrease in heart rate is primarily a reflex response to peripheral vasoconstriction and the consequent increase in systemic vascular resistance. In contrast, ketamine has indirect sympathomimetic properties associated with endogenous catecholamine release, which may contribute to the attenuation of bradycardia and the maintenance of hemodynamic stability. Notably, the Q3 group received only ketamine during the IM phase of the protocol, which may have resulted in more pronounced sympathomimetic effects until the subsequent intravenous administration of dexmedetomidine. This pharmacodynamic profile could have influenced cardiovascular parameters (13, 28, 31). This combination allowed deep sedation with minimal cardiovascular depression.

Mean heart rates observed in animals receiving dexmedetomidine were within an intermediate range when compared with available literature for the species and related taxa, being higher than those reported for xylazine-based protocols and similar to those observed with dissociative combinations containing α2-adrenergic agonists (4, 27, 30). These differences reinforce the influence of anesthetic protocol, route of administration, and body weight on heart rate, as increases in cardiac size and stroke volume reduce the need for higher heart rates to maintain cardiac output (26, 32).

The occurrence of second-degree AV block, Mobitz type II, in two animals that received IV dexmedetomidine is consistent with findings reported in small animals and humans, in which this drug is primarily associated with bradyarrhythmias and atrioventricular conduction disturbances (13, 18, 33). Until now, these effects had not been specifically described in white-lipped peccaries, highlighting the relevance of the present data.

Isolated premature supraventricular and ventricular complexes were also observed in animals receiving intravenous dexmedetomidine, alterations previously associated with increased vagal tone and reduced norepinephrine release induced by *α*2-adrenergic agonists (18, 34). The isolated occurrence of these arrhythmias without clinical repercussions suggests a transient pharmacological effect.

The reduced P wave amplitude observed in Q2 may reflect alterations in atrial depolarization associated with pharmacological factors, age, or individual characteristics. Similar values have been described in domestic pigs of comparable body weight and in small animals (26, 35, 36). Variations in T wave amplitude, which were greater in group Q3, may be related to ventricular repolarization and the action of α₂-adrenergic agonists, as well as factors such as body weight, pharmacological agents (e.g., barbiturates), and transient physiological conditions (18, 32, 37). The absence of significant electrolyte disturbances supports the pharmacological interpretation of these findings.

Another factor that may have contributed to the higher occurrence of arrhythmias in group Q3 is that these animals initially received ketamine alone. The literature describes that this dissociative anesthetic agent exerts positive inotropic effects, increasing myocardial oxygen consumption. In addition, it is associated with an increase in HR due to central nervous system stimulation, with enhanced sympathetic activity, which may increase the risk of arrhythmias (18, 25). Although ECG evaluation was performed after IV administration of dexmedetomidine in group Q3, the prior use of ketamine alone may have sensitized the cardiovascular system, favoring the occurrence of these alterations. In contrast, the other groups received pharmacological combinations from the outset, promoting greater synergism and sedative effect, which may have contributed to greater cardiovascular stability.

Animals receiving midazolam (Q1) did not present relevant alterations in cardiac rhythm or conduction, corroborating studies that describe this benzodiazepine as a drug with a stable cardiovascular profile (25, 32, 38, 39). Its association with ketamine resulted in heart rate values comparable to those previously described in collared peccaries, justifying its use as a comparative group with minimal cardiovascular interference. It is important to emphasize that the present study focuses on comparisons between different sedation protocols, rather than on comparisons with normal cardiovascular reference values, which are not established for this species. Additionally, the ketamine–midazolam combination has already been employed in the species for echocardiographic evaluation with the same objective of minimizing hemodynamic interference (7).

The data obtained in the present study demonstrate that the animals were subjected to a dissociative anesthetic protocol that ensured stability of vital parameters and data accuracy. This approach is consistent with studies emphasizing the need to perform echocardiographic examinations in wild mammals under anesthesia, given their propensity for stress and aggressive behavior. Anesthesia facilitates the procedure, minimizes stress, and ensures team safety; however, it should be acknowledged that anesthetic agents may induce changes in heart rate, blood pressure, and other hemodynamic parameters, potentially influencing the results (7, 40, 41).

Transthoracic echocardiography was performed under anesthesia, a condition considered appropriate for wildlife species due to reduced stress, immobility, and personnel safety, although it is acknowledged that anesthetic agents may influence hemodynamic parameters (7, 41). Images were predominantly obtained through parasternal windows, following established protocols for small animals, domestic pigs, and collared peccaries (7, 22, 26).

A relevant finding was the absence of morphological alterations in cardiac valves and other structures on echocardiography. Nevertheless, animals receiving dexmedetomidine showed reduced myocardial contractility compared with the other groups, possibly related to the negative chronotropic effect of the drug, including heart rate reduction, prolonged myocardial relaxation, and a potential increase in afterload, contributing to decreased cardiac output and contractile performance, as described in other studies (41, 42).

The LA, Ao, and LA/Ao ratio values were higher than those reported for collared peccaries (7), a finding primarily attributed to the greater body weight of the evaluated white-lipped peccary. Additionally, due to positioning and technical difficulties in obtaining echocardiographic images, it cannot be inferred that these values accurately reflect true measurements, and therefore they should not be considered reliable reference values. Nevertheless, the inclusion of only one individual reflects the practical challenges encountered during data acquisition in the remaining animals, which further underscores the need for cautious interpretation. Accordingly, although the LA/Ao ratio is commonly used as a reference parameter, its application in this context should be considered preliminary and interpreted with appropriate caution.

Similar differences were observed in ventricular dimensions, reinforcing the allometric principle widely described in mammals, according to which cardiac structures scale proportionally with body size (21, 43).

The only variable lower than that reported for collared peccaries was interventricular septal thickness in systole (IVSs), whereas the remaining measurements showed higher values, possibly reflecting morphofunctional adaptations related to the larger body size of white-lipped peccaries. The reduction in interventricular septal thickness in diastole (IVSd) observed in animals treated with dexmedetomidine may be associated with increased afterload and reduced cardiac output induced by this drug, as described by Poonia et al. (41).

Overall, echocardiographic measurements were similar to those described for dogs of comparable body weight but lower than those observed in domestic pigs within the same weight range, in which age exerted a significant influence on cardiac dimensions (26). In the present study, the absence of individual age records precluded a similar analysis.

Echocardiographic variables did not differ significantly among anesthetic protocols, indicating that the use of midazolam or dexmedetomidine did not compromise cardiac morphofunctional evaluation. Although dexmedetomidine induced bradycardia, this effect did not affect the reliability of echocardiographic measurements (13, 39, 41).

Given the use of different anesthetic protocols, the pooling of data in this study should be considered exploratory and interpreted with caution, as protocol-related effects may have influenced the cardiovascular variables evaluated.

Study limitations include difficulties in animal positioning and the absence of previously established echocardiographic standards for the species; furthermore, allometric normalization of the obtained data was not performed. Additionally, the concurrent execution of another project limited the collection of additional data, although examinations were performed by an experienced operator. All measurements were obtained under anesthesia and therefore reflect anesthesia-associated cardiovascular conditions rather than baseline physiological values; the absence of a conscious or minimally sedated control group further constrains interpretation, which was not feasible due to the species’ aggressive behavior and high sensitivity to handling. Nevertheless, the results presented contribute substantially to the understanding of the cardiovascular physiology of *Tayassu pecari* and provide a foundation for future clinical and conservation-oriented investigations.

## 5 Conclusion

In the absence of previously described electrocardiographic and echocardiographic reference values for white-lipped peccaries (*Tayassu pecari*), this study fills a relevant gap in the literature by establishing novel normative parameters for the species, with direct applicability to both clinical assessment and conservation management. The use of dissociative anesthetic protocols based on ketamine combined with dexmedetomidine or midazolam proved suitable for animal restraint, enabling stress reduction and the acquisition of reliable cardiovascular data.

The results indicate that dexmedetomidine is associated with cardiac electrophysiological changes, particularly related to rhythm and electrical conduction, in accordance with its central and peripheral sympatholytic effects. These alterations were not reflected in statistically significant differences in quantitative echocardiographic parameters among the evaluated anesthetic protocols.

Thus, the values established herein constitute a consistent initial preliminary reference for the species and represent a relevant advance in cardiovascular diagnosis, anesthetic management, and the development of conservation strategies for *Tayassu pecari*, while also providing a solid foundation for future studies in wildlife veterinary medicine.

## Data Availability

The original contributions presented in the study are included in the article/supplementary material, further inquiries can be directed to the corresponding author.
