# Correction: Electrocardiographic and echocardiographic profiles of white-lipped peccaries (*Tayassu pecari*) under ketamine–dexmedetomidine or ketamine–midazolam anesthesia

**DOI:** 10.3389/fvets.2026.1967366

**Published:** 2026-09-01

**Authors:** Rochelle Gorczak, Marilia Avila Valandro, Helena Bulhoes Barbosa, Raquel Vieira Niella, Mário Sérgio Lima de Lavor

**Affiliations:** 1 Department of Veterinary Medicine, Universidade Estadual de Santa Cruz, Ilhéus, Bahia, Brazil; 2 Independent Researcher, Porto Alegre, Brazil; 3 Department of Veterinary Medicine, Centro Universitário Ritter dos Reis, Porto Alegre, Rio Grande do Sul, Brazil

**Keywords:** cardiology, dissociative anesthesia, heart, hemodynamics, Tayassuidae, wild mammal

An incorrect **Funding** statement was provided. The correct funder is FAPESB (Fundação de Amparo à Pesquisa do Estado da Bahia) - APR0098/2026.

The correct **Funding** statement reads:

The author(s) declared that financial support was received for this work and/or its publication. This study was supported by Fundação de Amparo à Pesquisa do Estado da Bahia (FAPESB)-APR0098/2026.

The original version of this article has been updated.

